# Long-Term Mandatory Homeschooling during COVID-19 Had Compounding Mental Health Effects on Parents and Children

**DOI:** 10.3390/children11091072

**Published:** 2024-08-31

**Authors:** Lucretia V. M. Groff, Mariam M. Elgendi, Sherry H. Stewart, Storm Hélène Deacon

**Affiliations:** 1 Department of Psychology and Neuroscience, Dalhousie University, Halifax, NS B3H 4R2, Canada; lucretia.groff@dal.ca (L.V.M.G.); m.elgendi@dal.ca (M.M.E.); sherry.stewart@dal.ca (S.H.S.); 2 Department of Psychiatry, Dalhousie University, Halifax, NS B3H 4R2, Canada; 3 Department of Community Health and Epidemiology, Dalhousie University, Halifax, NS B3H 4R2, Canada

**Keywords:** COVID-19, homeschooling, parent mental health, child mental health

## Abstract

Background/Objectives: Most studies have linked mandatory homeschooling during COVID-19 to mental health harm in parents and children, while a minority have found non-significant or beneficial effects. Past studies have not measured mandatory homeschooling continuously over an extended period; consequently, they could not capture compounding mental health effects, which may explain conflicting results. We asked whether children’s cumulative time spent homeschooled during COVID-19 school closure mandates caused compounding harm for parent and child mental health, and whether parent employment, child internet access and educational support from schools (live and pre-recorded online classes, home learning packs) impacted this relationship. We aimed to identify the families at greatest risk of mental health harm during mandatory homeschooling and the educational support that may have mitigated this risk. Methods: Couples completed retrospective, cross-sectional survey questionnaires assessing parent depression, anxiety and stress, child internalizing and externalizing symptoms, and the family’s homeschooling experience. Data were analyzed using mediation analysis total effects, ordinary least squares regression and simple slopes analysis. Results: Both parents and children experienced compounding mental health harm during mandatory homeschooling. Live online classes protected parents and children, while home learning packs protected children. Unexpectedly, reliable internet access and the employment of both parents placed children at greater risk. Conclusions: Findings suggest that long-term mandatory homeschooling during COVID-19 placed families at greater risk of mental health harm. To protect family mental health during homeschooling mandates, schools should provide children with evidence-based educational support.

## 1. Introduction

On 11 March 2020, the World Health Organization declared the COVID-19 virus a pandemic [1]. To reduce transmission of the virus, over 90% of countries worldwide implemented school closures, forcing millions of children to continue their education through mandatory homeschooling [2]. Mandatory homeschooling refers to involuntary at-home learning resulting from mandated school closures, in which schooling responsibility is largely transferred onto parents and caregivers [3]. The COVID-19 pandemic has had negative impacts on parent and child mental health [4,5,6,7], which many studies have linked to mandatory homeschooling [3,8,9]. By contrast, a minority of studies have found that the effects of mandatory homeschooling on mental health were non-significant or beneficial [10,11]. 

Chronic (long-term) activation of stress response has been linked to greater adverse mental health impacts in adults and children than acute (short-lasting) stress [12,13,14,15]. These findings suggest that families engaged in long-term homeschooling may have been at greater risk of adverse mental health effects due to compounding (progressively worsening) effects over time [12,16]. However, existing research has not captured compounding mental health effects of mandatory homeschooling over an extended period, which may explain conflicting results across studies. Consequently, it is unknown whether mandatory homeschooling has compounding adverse mental health effects over time or if long-term mandatory homeschoolers are at greater risk.

Alongside the duration of mandatory homeschooling, several other factors may have influenced its effects on parent and child mental health. First, receiving inadequate educational support from schools during mandatary homeschooling may have placed additional academic strain on families, exacerbating mental health harm [17,18,19]. Second, since use of the internet was often necessary for mandatory homeschooling, families with poor internet access may have experienced greater mental health harm [20,21]. Finally, pre- and peri-pandemic research has linked parental unemployment to adverse mental health effects in families, which may point to parent unemployment as another risk factor during mandatory homeschooling [4,22,23,24].

The present study investigates whether children’s cumulative time engaged in mandatory homeschooling has compounding adverse effects on family mental health, looking specifically at parent depression, anxiety, and stress, and child internalizing symptoms (emotional and peer problems) and externalizing symptoms (behavioral and hyperactivity problems). It also explores whether educational supports from schools, child internet access or parent employment status moderate this relationship. Our first aim was to identify which families are at greatest risk of adverse mental health effects during mandatory homeschooling, so they can be given access to the supports they need to recover. Our second aim was to identify educational supports that may mitigate these adverse effects, to assist schools in minimizing mental health harm for families during future mandates.

### 1.1. Mandatory Homeschooling during COVID-19

During mandatory homeschooling, educational and childcare responsibilities usually fulfilled by schools were shifted onto parents, who often lacked adequate training and resources [25]. One study of mandated homeschooling during COVID-19 found that children spent 30% of their learning time in contact with their parents, while just under 14% was spent in contact with teachers [18]. In-person school provides children with academic support, structured routines, and access to mental health and peer supports, which are beneficial to children’s mental health [26,27]. However, during the COVID-19 school closures, children were cut off from many of these mental health protections [19,27]. Meanwhile, many parents were forced to navigate their newfound schooling responsibilities alongside other stressors, including employment insecurity, financial strain, and social inequities [4,7].

### 1.2. Mandatory Homeschooling and Parent and Child Mental Health 

Most research examining mandatory homeschooling during the COVID-19 pandemic has linked it to declines in parent and child mental health. Kishida et al. [8] found that full school closures the previous week were linked to greater parent and child mental health harm compared to partial school closures, which in turn were linked to greater harm than full in-person learning. Schmidt et al. [9] found that parents and children experienced poorer affect on mandatory homeschooling days, while Deacon et al. [1] found that parents who spent more hours per week homeschooling their child due to COVID-19 mandates experienced higher anxiety, depression, and traumatic stress. Interestingly, a minority of studies did not support these findings. DesRoches [10] found that mandatory homeschooling had non-significant impacts on child mental health, while Monnier [11] found that more hours per day spent homeschooling were associated with lower externalizing symptoms in children. This lack of consensus on the effects of mandatory homeschooling on child mental health may be explained by how these studies conceptualized and measured mandatory homeschooling.

### 1.3. Homeschooling Time as a Continuous Measure

The duration of school closures during the COVID-19 pandemic varied by location and school board [28]. In Canada, school closure duration ranged from a minimum of 50 school days in British Columbia, to a minimum of 135 school days in Ontario [29]. Past research has studied mandatory homeschooling over short-term periods, often using non-continuous measures that do not capture variations in duration [3,8,9,10,11]. For example, some studies have measured mandatory homeschooling as a dichotomous yes/no variables [9,10], while others have measured it as the amount of time spent homeschooling per week [3,8] or day [11]. Consequently, these studies are unable to differentiate between families who experienced long-term versus short-term mandatory homeschooling. 

Stressors are actual or perceived threats to an organism that provoke physiological, emotional and/or behavioral reactions from the body. These reactions, collectively called the stress response, prepare the body to cope with stressors [16]. Chronic (long-term) activation of the stress response has been linked to mental health problems in children and adults, including depression, anxiety and behavioral problems, which compound over time [12,13,14,15]. 

Based on findings that chronic stress causes compounding mental health harm over time, it is possible that families engaged in long-term mandatory homeschooling experienced greater mental health harm than those engaged in short-term homeschooling. Further, short-term homeschooling may have had positive mental health impacts on some families by providing increased opportunities for family support and removing children from potential school stressors such as bullying [30,31]. Because past research used non-continuous measures of mandatory homeschooling, the findings could not capture potential compounding mental health effects over time; this may account for the conflicting results on the effects of mandatory homeschooling. To our knowledge, the present study is the first to measure time spent engaging in mandated homeschooling continuously over an extended period. Consequently, it is unknown whether mandatory homeschooling has compounding negative mental health effects that place long-term homeschoolers at greater risk. 

### 1.4. Educational Support from Schools

Throughout the COVID-19 pandemic, many countries provided minimal national guidance about which educational support should be provided by schools [32]. Consequently, there was wide variability in the support children received from their schools or governments [20,32]. Many families attributed mandatory homeschooling challenges during the COVID-19 pandemic to a lack of educational resources and support from schools [17,33], the latter of which was associated with greater parental distress [34]. Given these findings, it is possible that receiving educational support from school may have benefited mental health during mandatory homeschooling. 

Many children received online classes from schools during mandated homeschooling [20]. Pre-recorded online classes may have provided students with scheduling flexibility and higher-quality presentations, while live online classes may have provided opportunities for live student–teacher interaction and questions [35,36]. Live online classes may have been especially beneficial for mental health, given findings that children who interacted more with their teacher found online learning more effective and were more satisfied with their experience [19]; however, not all students had access to online classes during mandated homeschooling [18,20]. It is possible that children struggled more when they did not receive online classes, especially live ones, resulting in greater mental health harm for families during mandatory homeschooling. 

Many students also received other digital or physical resources such as assignments, worksheets or videos, which can be conceptualized as home learning packs [20,32,37,38]. Home learning packs may have likewise contributed to improved mental health during mandatory homeschooling by providing children with a flexible opportunity to supplement and apply knowledge they gained in class. Pre-pandemic research suggests that these opportunities may have improved children’s academic experiences, suggesting that home learning packs may have decreased academic strain on families during mandatory homeschooling [39,40]. This study examines live and pre-recorded online classes, as well as home learning packs, as educational supports from schools that may have mitigated adverse mental health effects of mandatory homeschooling.

### 1.5. Child Internet Connection

While specific school supports varied during mandated homeschooling, most children had an online component to education during the pandemic [20], making reliable internet connectivity a crucial resource. Unreliable internet connection has been linked to distress and dissatisfaction during mandatory homeschooling [17,41], which may suggest that it contributed to families’ mental health harm. Despite its importance, many children struggled to access reliable internet connection during the pandemic in Canada and the United States, with some children relying entirely on public Wi-Fi to complete homework [21,42]. Alternatively, it is possible that reliable internet access put children at greater risk of problematic internet use, which increased during the pandemic and has been linked to poor mental health in children [43,44]. Together, the prevalence of unreliable internet access and its potential to harm (or benefit) family mental health during mandatory homeschooling prompted us to investigate its effects in the present study.

### 1.6. Parent Employment

Many parents either lost their jobs during the COVID-19 pandemic or reduced their working hours to care for their children [45]. Findings are mixed regarding the effects of parent employment on family mental health during COVID-19. Pre-pandemic research has linked self- or spousal unemployment to stress, anxiety and depression in adults [23,46,47]. Some pre-pandemic studies have linked parental unemployment to poorer mental health outcomes for children due to family financial strain and reduced parental well-being [24], while others have found beneficial impacts on younger children due to increased availability of parent–child quality time [48]. In line with many pre-pandemic findings, parent unemployment during the pandemic has been associated with increased depression in parents [5] and increased negative affect in children [49]. Employed parents, however, reported that balancing the responsibility of their child’s education with work was a major source of stress, and many felt unable to provide their child with adequate school support [4,7,22]. These findings prompted us to explore parent employment status as a factor that may have influenced family mental health during mandatory homeschooling.

### 1.7. Current Study

The goal of the present study is to assess the mental health impact of children’s time spent engaged in mandated homeschooling during the COVID-19 pandemic for children and parents. We measure mandatory homeschooling continuously, as the percentage of time between September 2020 and 15 February 2021. In line with prior research, we conceptualize child mental health as internalizing symptoms and externalizing symptoms and parent mental health as depression, anxiety, and general stress [3,11,50]. The study further assesses whether this relationship is moderated by parent employment status, child internet access, or educational support from the child’s school, including live online classes, pre-recorded classes, or home learning packs (e.g., worksheets, assignments, videos, etc.).

This study answers recent calls for research into the effects of COVID-19 restrictions on child and parent mental health [51]. By studying the compounding mental health effects of cumulative mandatory homeschooling over time, we aim to clarify contradictory findings in past research. By further exploring which factors may moderate this relationship, we aim to identify the families at greatest risk of adverse mental health effects during mandatory homeschooling and the educational support mitigating these effects. Our results may help governments and schools connect high-risk families with the support they need to recover from lingering mental health effects of the COVID-19 pandemic and may encourage schools to implement effective educational support in the future. Together, our results may shape how mandatory homeschooling is conceptualized in future research to capture compounding mental health effects. They may also guide policy aimed at minimizing mental health harm when implementing future homeschooling mandates, such as limiting duration. This study assesses the following hypotheses.

**Hypothesis 1 (H1).**  *We predict that cumulative time spent homeschooling is positively associated with child internalizing and externalizing symptoms and parent anxiety, depression and stress, such that greater cumulative time spent homeschooling is associated with poorer mental health.*

**Hypothesis 2a (H2a).**  *We predict that live online class moderate this relationship, such that receiving this educational support will predict decreased mental health harm of cumulative time spent homeschooling for parents and children.*

**Hypothesis 2b (H2b).**  *We predict that pre-recorded online classes moderate this relationship, such that receiving this educational support will predict decreased mental health harm of cumulative time spent homeschooling for parents and children.*

**Hypothesis 2c (H2c).**  *We predict that home learning packs moderate this relationship, such that receiving this educational support will predict decreased mental health harm of cumulative time spent homeschooling for parents and children.*

**Hypothesis 3 (H3).**  *We predict that children’s internet access moderates this relationship, such that unreliable internet access will predict increased mental health harm from cumulative time spent homeschooling for parents and children.*

**Hypothesis 4 (H4).**  *We predict that parent employment moderates this relationship, such that unemployment of one or both parents will predict greater mental health harm from cumulative time spent homeschooling for parents and children.*

## 2. Materials and Methods

This study is based on an archival data set from the study COVID-19 Pandemic: Factors that Support and Impede Family Well-being During Mandatory Homeschooling conducted by the Language and Literacy Lab and the Mood, Anxiety and Co-Morbidity Lab at Dalhousie University.

### 2.1. Participants

Participants in this study were 718 romantic partners with at least one school-aged child in grades 1–5 (Table 1). Data were collected about each family’s youngest school-aged child. Of these children, 332 were undergoing full- or part-time mandatory homeschooling due to COVID-19 mandates, while the remaining 338 were being schooled in person full time. Study eligibility required both parents to be at least 19 years old and living in Canada or the U.S. They had to have lived together during the COVID-19 pandemic and have been in a romantic relationship with each other for at least 3 months preceding the study. They also had to have at least one child in grades 1–5 involved in either mandatory homeschooling or full-time in-person learning between September 2020 and 15 February 2021.

### 2.2. Procedure

After receiving approval from the Dalhousie University Research Ethics Board (#2020-5166), data collection for this study took place between 18 March and 18 May 2021. Couples were recruited via Qualtrics Survey Panels and provided data at a single time-point. After eligibility screenings, both members of eligible couples provided informed consent to participate. Via Qualtrics, each parent retrospectively reported on their own demographic information and mental health in the 30-day period between 15 January and 15 February (symptoms of depression, anxiety, and stress). One parent (called Parent A) reported on their youngest school-aged child, including the child’s demographic information, mental health (internalizing and externalizing symptoms), the homeschooling support they received from school, and the percentage of time the child was engaged in mandated homeschooling between September 2020 and February 2021. The surveys also included attention-check questions and response-speed verifications to ensure that participants were considering their responses, and those who failed either verification were excluded. The 718 couples in the sample all passed verification. Participants were each compensated with one USD 10 Amazon gift card.

### 2.3. Measures

#### 2.3.1. Demographics Questionnaire

Parents reported their age, gender, sex, race/ethnicity, relationship, income, education level, and employment status. Parent A reported their youngest school-aged child’s age, gender, and race/ethnicity (Table 1).

#### 2.3.2. Cumulative Time Spent Homeschooling

Cumulative time spent homeschooling was measured as the parent-reported percentage of time children were engaged in mandatory homeschooling between September 2020 and 15 February 2021. This period was chosen because it captures a significant wave of the COVID-19 pandemic, during which mandatory homeschooling and in-person learning were co-occurring across the U.S. and Canada [52].

#### 2.3.3. Child Internalizing and Externalizing Symptoms

Child mental health symptoms were measured using the internalizing and externalizing subscales of the Strengths and Difficulties Questionnaire (SDQ), which can be completed by parents of 4–16-year-olds [50]. The SDQ consists of five subscales: emotional problems, peer problems, behavioral problems, hyperactivity, and prosocial behavior. The 10-item Child Internalizing Subscale (Cronbach’s *α* = 0.83) combines the emotional problems (e.g., “Many worries or often seems worried”) and the peer-related problems (e.g., “Picked on or bullied”) subscales, while the 10-item Child Externalizing Subscale (Cronbach’s *α* = 0.79) combines the behavioral problems (e.g., “Often loses temper”) and hyperactive problems, (e.g., “Easily distracted”) subscales [50]. Items from the SDQ are measured on a 3-point response scale (“Not true” = 0, “Somewhat true” = 1, “Certainly true” = 2), and scores for each subscale can range from 1 to 20, with higher scores representing greater presence of symptoms. In this version of the SDQ, parents were asked to what extent statements were true of their child within the 30-day period between 15 January and 15 February 2021. The SDQ has good internal consistency (Cronbach’s *α* = 0.73) [53] and good convergent and discriminant validity [50].

#### 2.3.4. Parent Depression

Parent depression was measured using the 9-item Patient Health Questionnaire (PHQ-9; Parent A Cronbach’s *α* = 0.92; Parent B Cronbach’s *α* = 0.91), a self-report survey which assesses the DSM criteria for depression on a 4-point scale (0 = “not at all”, 3 = “everyday”) [54]. Scores on the PHQ-9 range from 0 to 27, with greater scores representing greater depressive symptoms. Scores from both parents were summed into a single couple score to capture collective parent depression. This creation of a composite score across parents is similar to the approach we used in past studies with couples to capture a dyadic level variable [55,56]. Depression scores between Parent A and Parent B were highly correlated (r = 0.49, *p* ≤ 0.001), providing psychometric support for this approach. The PHQ-9 asks how frequently the respondent has been bothered by a symptom in the past two weeks (e.g., “Feeling down, depressed or hopeless”). In the current study, this time frame was modified to include the 30-day period between 15 January and 15 February 2021 to better capture the period of ongoing homeschooling. The PHQ-9 has shown good internal reliability (Cronbach’s *α* = 0.89), criterion validity, and construct validity in adult patients [54].

#### 2.3.5. Parent Anxiety

Parent anxiety was measured using the 7-item General Anxiety Disorder scale (GAD-7; Parent A Cronbach’s α = 0.92; Parent B Cronbach’s α = 0.92), a self-report survey which assesses anxiety symptoms (e.g., “Worrying too much about different things”) on a 4-point scale (0 = “not at all”, 3 = “nearly every day”) [57]. Possible scores ranged from 1 to 27, with greater scores indicating higher levels of anxiety. Parent scores were summed into one collective anxiety score, which is similar to the approach we used in past studies with couples to capture a dyadic level variable [55,56]. The high correlation (r = 0.45, *p* = <0.001) between Parent A and Parent B provides psychometric support for this approach. The GAD-7 asks how frequently the respondent has been bothered by a symptom in the past two weeks, which was modified in the current study to capture the 30-day period between 15 January and 15 February 2021, a longer period of ongoing homeschooling. The GAD-7 has good internal consistency (Cronbach’s *α* = 0.92) and good criterion and construct validity in adult patients [57].

#### 2.3.6. Parent Stress

Parent stress was measured using the 4-item Perceived Stress Scale (PSS-4; Parent A Cronbach’s *α* = 0.64; Parent B Cronbach’s *α* = 0.59), a self-report survey which assesses stress symptoms on a 5-point scale (0 = “Never”, 4 = “Very Often”) [58]. The PSS-4 asks how frequently the respondent has experienced a symptom in the last month, which we modified to capture the 30-day between 15 January and 15 February 2021 (e.g., “Feeling unable to control the important things in life”). Possible scores on the PSS range from 0 to 28, with greater scores indicating greater levels of stress. Both parents’ scores were summed into a single couple score, representing their collective stress. In past studies with couples, we used a similar approach to capture a dyadic level variable [55,56]. Parent A and Parent B stress scores were highly correlated (r = 0.5, *p* ≤ 0.001), which provides psychometric support for this approach. The PSS-4 has good internal consistency (Cronbach’s *α* = 0.77) [58] and good validity in adult populations [59].

#### 2.3.7. Educational Support from Schools

Online live class. Parent A reported whether their child’s school provided live online classes (e.g., yes or no).

Online pre-recorded class. Parent A reported whether their child’s school provided pre-recorded online classes (e.g., yes or no).

Home learning packs. Parent A reported whether their child’s school provided home learning packs (e.g., yes or no). Examples of home learning packs given to participants included “worksheets, assignments, videos, etc.”.

#### 2.3.8. Child Internet Connection

Children’s access to reliable internet connection was measured using a 7-point Likert scale (0 = “not at all”, 6 = “very much”). Parent A responded to the following question: “Between 15 January and 15 February, to what extent did your child have access to a reliable internet connection?”. Internet connection was then re-coded into a binary variable to account for inadequate sample sizes in some response categories, such that Reliable Internet included scores of 6 and Unreliable Internet included scores from 0 to 5.

#### 2.3.9. Parent Employment

Parent employment status was coded as Unemployed vs. Employed. The employed category included those employed full and part time, while the unemployed category included those who were unemployed (e.g., looking for work) or not in the labor force (e.g., not performing or looking for paid work). We combined unemployed people with those not in the labor force due to low sample sizes in some categories and because distinctions between these groups were blurred during the pandemic due to the volatile state of the labor force [60] and increases in unpaid care-giving labor [61]. We combined both parents into a single variable, coded as Both Employed vs. One or Both Unemployed, to capture compounding effects of parental employment.

### 2.4. Analysis Plan

We conducted statistical analyses for this study using version 4.2.2 of R [62]. Assumption screening revealed violation of normality, which was unlikely to impact our results due to our large sample size (>10 observations per variable) [63,64]. There was further violation of homogeneity of variance, which we addressed by conducting heteroskedasticity-consistent standard errors using PROCESS macro for R version 4.3.1 [65,66]. PROCESS macro is a modeling tool used for path analysis with ordinary least squares and logistic regressions [67]. It can be used to test two-way moderation models, simple slopes, and mediation models while incorporating heteroskedasticity-consistent standard errors [65,67]. Significance was determined using an alpha level of *p* < 0.05. Continuous variables were grand-mean-centered, and categorical variables were effect-coded.

**Figure 1:**
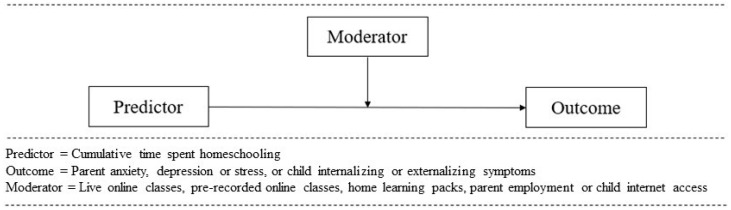
Conceptual Model for Ordinary Least Squares Regression. Note. This conceptual model demonstrates the intention of the ordinary least squares regressions to determine whether the relationship between cumulative time spent homeschooling and the mental health outcome is changed by the moderator. This image is based on models from Moon [68] and Hayes [66].

We used mediation models to assess the total effects of cumulative time spent homeschooling on parent and child mental health (H1). This approach was used because PROCESS macro does not support basic linear regression. Using mediation models to extract the total effects of the predictor on the outcomes enabled us to use the same software for all our models, thus maintaining consistency across our analyses. Next, ordinary least squares regression and simple slopes analysis were used to test whether the relationships between cumulative time spent homeschooling and parent and child mental health are moderated by parent employment status, children’s internet access, live online instructions, pre-recorded online instruction, or home learning packs (H2–H4). Ordinary least squares regressions tested for moderation by assessing whether interactions between cumulative time spent homeschooling and the moderators accounted for variation in the mental health outcomes, as indicated by significant (*p* < 0.05) changes in R^2^ (Figure 1). For significant interactions, simple slopes analyses were used to assess the relationship between cumulative time spent homeschooling and mental health outcomes at different levels of the moderator to determine whether they supported our hypotheses. We ran one mediation model and four ordinary least squares regressions for each of our mental health outcomes (25 total).

## 3. Results

Demographic information and means for study variables are presented in Table 1 and Table 2, respectively.

### 3.1. Cumulative Time Spent Homeschooling and Parent and Child Mental Health (H1)

Shown in Table 3, total effects from mediation models revealed that child internalizing symptoms, parent anxiety, and parent depression were all significantly and positively associated with time spent homeschooling. Child externalizing symptoms and parent stress were not significantly associated with cumulative time spent homeschooling.

### 3.2. Live Online Classes as a Moderator (H2A)

**Figure 2:**
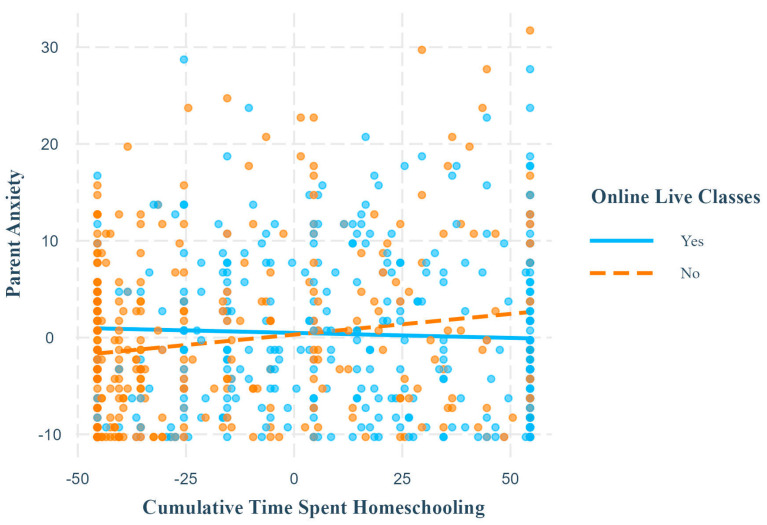
Simple slopes for parent anxiety outcome, online live classes moderator. Note. This plot shows the relationship between cumulative time spent homeschooling and parent anxiety at different levels of the moderator, online live classes. The relationship was significant and positive when children did not receive online classes but was non-significant when they did. Axis numerical ranges reflect grand mean centering or effect coding of variables.

Ordinary least squares regressions revealed that live online classes significantly interacted with cumulative time spent homeschooling to predict child internalizing symptoms and parent anxiety and depression, but not parent stress or child externalizing symptoms (Table 4). Simple slopes probing revealed that cumulative time spent homeschooling was only significantly positively associated with child internalizing symptoms, parent anxiety (Figure 2), and parent depression when children did not receive live online classes. There was no significant association when children did receive live online classes (Table 5).

### 3.3. Pre-Recorded Online Classes as a Moderator (H2B)

Ordinary least squares regressions revealed that pre-recorded online classes did not significantly interact with cumulative time spent homeschooling for any of the mental health outcomes (Table 4).

### 3.4. Home Learning Packs as a Moderator (H2C)

**Figure 3:**
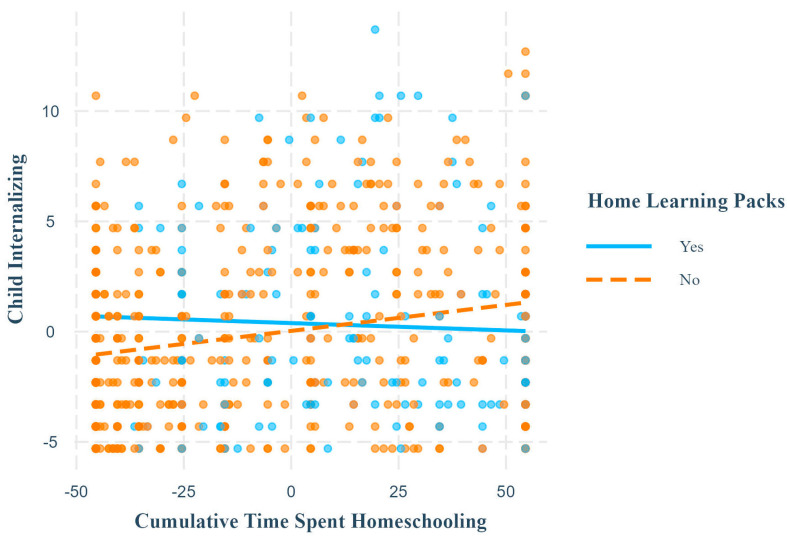
Simple slopes for child internalizing outcome, home learning pack moderator. Note. This plot shows the relationship between cumulative time spent homeschooling and child internalizing symptoms at different levels of the moderator, home learning packs. The relationship was significant and positive when children did not receive online classes but was non-significant when they did. Axis numerical ranges reflect grand mean centering or effect coding of variables.

Ordinary least squares regressions revealed that home learning packs significantly interacted with cumulative time spent homeschooling to predict child internalizing symptoms and parent depression (Table 4). Simple slopes probing revealed that these relationships were significant and positive only when children did not receive home learning packs (Table 5; Figure 3).

### 3.5. Internet Connection as a Moderator (H3)

**Figure 4:**
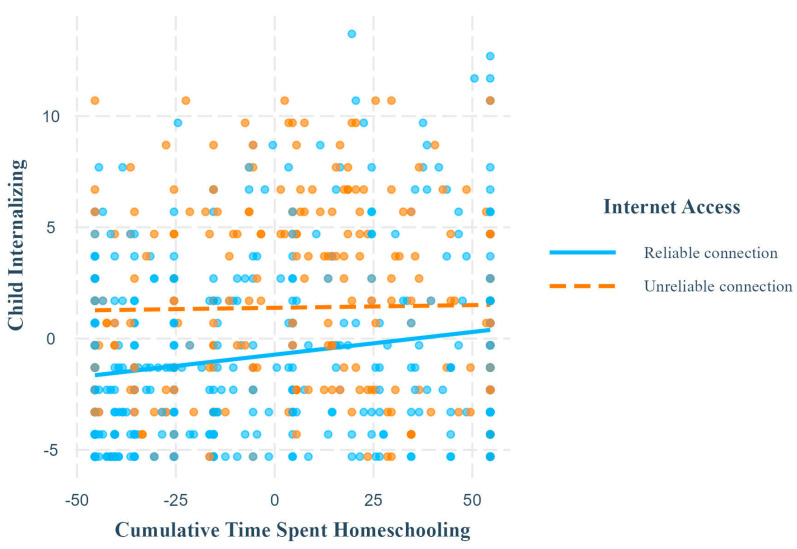
Simple slopes for child internalizing outcome, internet access moderator. Note. This plot shows the relationship between cumulative time spent homeschooling and child internalizing symptoms at different levels of the moderator, internet access. The relationship was significant and positive when they had reliable internet access but was non-significant when they had unreliable internet access. Axis numerical ranges reflect grand mean centering or effect coding of variables.

Ordinary least squares regressions revealed that internet connection interacted with cumulative time spent homeschooling to predict child internalizing symptoms (Table 6). Simple slopes revealed that cumulative time spent homeschooling was significantly positively associated with child internalizing symptoms when children had access to reliable internet connection, but not when they had unreliable internet connection (Table 5; Figure 4).

### 3.6. Parent Employment as a Moderator (H4)

**Figure 5:**
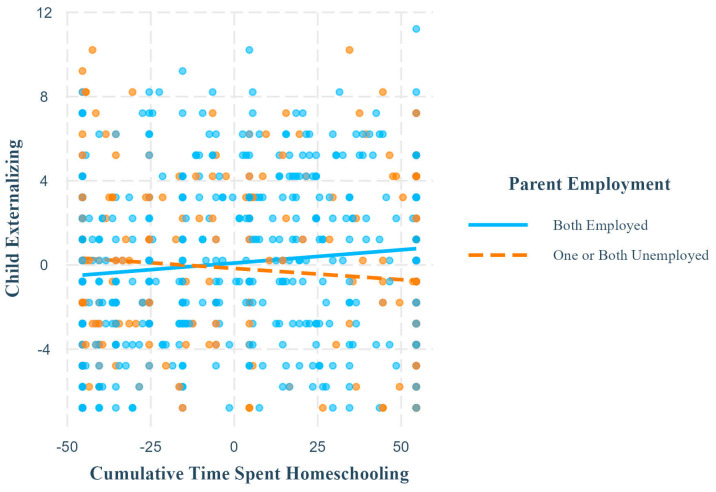
Simple slopes for child externalizing outcome, parent employment moderator. Note. This plot shows the relationship between cumulative time spent homeschooling and child internalizing symptoms at different levels of the moderator, parent employment. The relationship was significant and positive when both parents were employed but was non-significant when one or both were unemployed. Axis numerical ranges reflect grand mean centering or effect coding of variables.

Ordinary least squares regressions revealed that parent employment interacted significantly with cumulative time spent homeschooling to predict child internalizing and externalizing symptoms (Table 6). Simple slopes analysis revealed that cumulative time spent homeschooling was significantly positively associated with child externalizing (Figure 5) and internalizing symptoms when parents were employed, but not when one or both parents were unemployed (Table 5).

## 4. Discussion

In the present study, we explored compounding adverse mental health effects of mandatory homeschooling for families during the COVID-19 pandemic by assessing parent and child mental health and children’s cumulative time spent homeschooling over several months. We further explored the effects of potential risk and protective factors on this relationship, including educational support from schools, child internet access, and parent employment.

We found that greater cumulative time spent homeschooling was significantly associated with greater child internalizing symptoms and parent depression and anxiety, but not child externalizing symptoms or parent stress, showing partial support for H1. This supports findings from most past research linking mandatory homeschooling to adverse mental health effects for parents and children [3,8,9]. It also extends this research by suggesting that these effects compound over time, placing long-term homeschoolers at greater risk of mental health harm. These findings align with chronic stress research, which shows that long-term exposure to stressors (such as mandatory homeschooling) is harmful for adult and child mental health [12,13,14,15]. These findings also suggest that measuring mandatory homeschooling continuously over longer periods may be a more effective means of investigating its effects on mental health.

Our results also suggest that the effects of mandated homeschooling may be more salient for some mental health outcomes than others. The non-significant association between cumulative time spent homeschooling and child externalizing symptoms may relate to findings by Monnier et al. [11], which show that three or more hours spent homeschooling per day improved children’s hyperactivity symptoms. Research has found that daily routines, which could be built around homeschooling tasks, may have had positive impacts on children’s externalizing symptoms [10,69]. There may exist a “sweet spot” of time spent homeschooling during school closure mandates that is protective for children’s externalizing symptoms, which would not be captured in the total effects of our mediation models. The non-significant relationship between cumulative time spent homeschooling and parent stress may be explained by our measure of stress, the PSS-4, which captures general stress [58]. During mandatory homeschooling, parents may have been experiencing forms of stress specific to the COVID-19 pandemic, which may not have been captured by this measure. A stress measure specific to COVID-19, such as the COVID-19 Stress Scale developed by Taylor et al. [70], may therefore be more appropriate. Deacon et al. [1] used this scale to find that greater time engaged in mandatory homeschooling was linked to increased COVID-19-related stress in parents, supporting this possibility.

Looking at educational support provided by schools (H2)**,** we found that live online classes (H2a) and home learning packs (H2c) but not pre-recorded online classes (H2b) moderated the relationships between cumulative time spent homeschooling and some mental health outcomes. First, we found that cumulative time spent homeschooling was only positively associated with child internalizing symptoms and parent depression and anxiety when children did not receive live online classes. These findings may reflect the importance of live student–teacher interactions for academic success and satisfaction during mandatory homeschooling [19]. Live classes may have allowed children to depend more on teachers for ongoing academic support, easing the educational strain felt by families when tackling academic and technological challenges at home [25,33]. Live online classes also create routine in the child’s day, which has been identified as a protective factor for children’s mental health during COVID-19 [10,69]. This routine may have helped parents by offering them structured time to focus on other responsibilities while children are occupied by teachers. By contrast, pre-recorded online classes did not offer live academic support or routine, which may explain their non-significant interaction effects. Instead, children learning from pre-recorded classes may have sought support from parents, adding to the family’s academic strain. The added responsibility of scheduling class time may have also fallen to parents.

We also found that home learning packs protected against the effects of cumulative time spent homeschooling on child internalizing symptoms and parent depression. The relationships between mandatory homeschooling and child internalizing symptoms or parent depression were only significant and positive when children did not receive home learning packs. This suggests that children and parents benefitted when children received educational support such as assignments, worksheets, or videos during mandated homeschooling. For parents, having additional resources to rely on may have helped them instruct their children more effectively while sparing them the additional effort of assembling materials on their own. This may have protected parents by reducing the burden of their newfound educator roles. These resources may also have provided children with opportunities to learn independently and apply their knowledge, which has been linked to improved academic performance [40]. If home learning packs helped students and parents engage with course content more effectively and overcome academic challenges, this may explain their protective effects on child mental health. Together, these results suggest that educational support for schools, especially live online class and home learning packs, protect families against the adverse mental health impact of mandatory homeschooling.

Our results suggest that reliable internet access is a risk factor for children’s mental health during mandatory homeschooling, contrary to our hypothesis (H3). Cumulative time spent homeschooling was only significantly positively associated with child internalizing symptoms when children had reliable internet access. We hypothesized that reliable internet connection would protect mental health because of its crucial importance for school, work, social connection, entertainment, and health care during the pandemic [71,72]. However, excessive internet use before and during COVID-19 has been linked to adverse mental health and academic outcomes in children [25,44,73,74]. Further, problematic internet use among children, including elevated time online, increased throughout the COVID-19 pandemic [43,44]. Therefore, children with reliable internet may have been using the internet excessively or in otherwise problematic ways, which may in turn have interfered with their schooling and had adverse impacts on their mental health. By contrast, when children were distracted by the internet, parents’ mental health may have benefitted from having time to themselves, explaining why adverse effects did not extend to parents.

Finally, our results suggest that having both parents employed is a risk factor for child mental health during mandatory homeschooling, contrary to our hypothesis (H4). There was only a significant positive relationship between cumulative time spent homeschooling and child internalizing and externalizing symptoms when both parents were employed. These findings suggest that cumulative time spent homeschooling only has adverse impacts on child mental health when both parents are employed. This finding may reflect reports that balancing work and homeschooling is a major source of stress for employed parents [4,7,22] and past findings that children show more internalizing and externalizing symptoms when parents experience greater work–family conflict [75]. It is possible that working parents had less time to support their children academically, which may have caused their children to struggle more with school and consequently experience mental health declines. By contrast, unemployed parents may have had more time and energy to support their children with school. For parents, the unique challenges of being unemployed during a pandemic may have negated the benefits experienced by their children, explaining the non-significant results for parent mental health [4,7,22]. These findings suggest that the effects of unemployment differed in the context of the COVID-19 pandemic, and that families with two working parents may have been at greater risk of mental health harm.

### Limitations and Future Directions

Our study includes some limitations. First, our data were collected at a single time point and accounts were retrospective, preventing causal conclusions. Parents and children were mostly white (parent = 69%, children = 67%), parent relationships were mostly heterosexual (94%), and only 14% of families had incomes of USD 50,000 or less. It is important that future research include more diverse samples, given that racial, ethnic, and sexual minorities and people of lower socioeconomic status experienced disproportionate challenges during the COVID-19 pandemic due to inequities [4,76]. Given the disproportionate impacts experienced by people of lower socioeconomic status, we ran additional sensitivity tests using family income (adjusted for family size) as a covariate. Our results unchanged, suggesting that the effects of family income on mental health likely do not account for our results; however, future research should continue to include relevant sociodemographic variables as covariates in their models.

Another limitation is that we used binary yes/no measures for educational support from school and did not collect information on its quality or the training provided to families for this support. Families reported that poor-quality educational support and lack of training on how to use it contributed to adverse mandatory homeschooling experiences [25,37], suggesting that these factors should be explored in future research.

This study also ran multiple (30 total) tests, which can increase the risk of Type 1 error. A stringent method to protect against Type 1 error with multiplicity testing is the Bonferroni correction, which is calculated by dividing the standard significance threshold of *p* < 0.05 by the number of tests [77]—in our case, 30. With a stringent Bonferroni-corrected alpha of 0.0017 (0.05/30), only one of our findings remained significant, namely the positive relationship between time spent homeschooling and child internalizing symptoms (*p* < 0.001). Thus, we can have confidence in the observed positive link of time spent homeschooling with child internalizing symptoms. Other findings reported as statistically significant in the main analyses using traditional significance levels (*p* < 0.05) should be interpreted with caution and must be replicated in future studies. Our survey data were also collected at a single time point, which limits our ability to determine causality and temporality [78]. While our cross-sectional design is a useful first step, further studies using longitudinal designs are needed to replicate our findings and confirm causality [79]. Our findings therefore represent important early findings.

Our conceptualization of parent employment also presents limitations. We combined “Unemployed” parents with those “Not in the labor force” to account for low sample sizes, based on findings that these employment statuses resembled each other during the pandemic [60,61]. However, these categories are usually viewed as distinct from each other, and should also be explored separately in future research to determine whether their effects differ [80]. To further account for the small sample size of families in which both parents were unemployed (*n* = 35), the “Unemployed” category combined families with one and two unemployed parents. This may present a limitation, as experiences of families with two unemployed parents may have differed from those with one employed and one unemployed parent [81]. For example, the latter families may have benefited from having one parent with stable employment and one parent with extra time to support the child’s homeschooling [82], which could have influenced our results. To address this limitation, we ran supplementary analyses that assessed the parent employment moderator at three levels: Both Employed, Both Unemployed and Mixed (One Employed, one Unemployed). Results were virtually unchanged, suggesting that combining these groups did not adversely affect our results. However, future studies should still consider exploring potential differences between families with one versus two unemployed parents.

Past research has also found that mothers took on a disproportionate share of childcare responsibilities during the pandemic compared to fathers and may have experienced more adverse mental health effects [83,84]. Further, paternal and maternal unemployment may impact child mental health differently [48]. Considering these findings, future studies should also assess differences in the effects of maternal versus paternal employment on family mental health during COVID-19. Future studies should also explore whether families with multiple school-aged children experienced mandatory homeschooling differently.

## 5. Conclusions

The present study investigated the compounding mental health impacts of cumulative time spent homeschooling for parents and children and sought to identify relevant risk and protective factors that may moderate these relationships. Our study extends past research on mental health during mandatory homeschooling by demonstrating that cumulative mandatory homeschooling had compounding adverse effects on mental health over time, which suggests that families engaged in long-term mandatory homeschooling were at greater risk. These findings may prompt policy makers to limit the duration of future homeschooling mandates or to explore alternatives instead. They also suggest that measuring mandatory homeschooling as a continuous variable over time may be a more effective strategy for studying its effects on mental health, which future researchers should consider adopting.

Our results also suggest that the cumulative mental health harm faced by families during mandatory homeschooling was exacerbated when both parents were employed. These families may require additional mental health and academic support to recover from the effects of mandatory homeschooling. Teachers and schools should work with affected students and their families to develop plans for recovery. Schools and governments should likewise consider subsidizing tutoring support programs for families that may be struggling with lingering impacts of mandatory homeschooling. Finally, our results highlight the crucial role schools and teachers play in maintaining family mental health during mandatory homeschooling. Effective support from schools, especially in the form of live online classes, appears hugely impactful in improving mental health outcomes for families. Schools should provide live online classes and other evidence-based educational support as a required component of education during mandatory homeschooling. School boards must likewise ensure that teachers, parents, and students all receive access to the technologies and training needed for these educational approaches to be implemented effectively.

## Figures and Tables

**Table 1:** Demographic information for parents and children.

Variable	Families (*n* = 718)
Country	
Canada	647 (90%)
U.S.	71 (10%)
Parent Age—*M* (*SD*)	39 (6.4)
Parent Gender	
Female	711 (<49.5%)
Male	722 (50.3%)
Non-binary/Unknown	3 (0.002%)
Parent Relationship Structure	
Mixed Sex	678 (94.4%)
Same Sex	27 (3.8%)
Other/Prefer Not to Answer	3 (0.004%)
Family Income	
USD 25,000 or less per year	30 (4.2%)
Between USD 26,000 and USD 50,000	74 (10.3%)
Between USD 51,000 and USD 75,000	103 (14.3%)
Between USD 76,000 and USD 100,000	140 (19.5%)
Between USD 101,000 and USD 125,000	102 (14.2%)
Between USD 126,000 and USD 150,000	109 (15.2%)
USD 151,000 or more per year	132 (18.4%)
Prefer not to answer	28 (3.9%)
Parent Highest Level of Education	
Some High School	32 (2.2%)
High School Graduate	155 (10.8%)
Some College/University	155 (10.8%)
College/University Graduate	715 (49.8%)
Some Post-Graduate	75 (5.2)
Post-Graduate Degree	304 (21.2%)
Parent Employment Status	
Employed	1170 (81.5%)
Employed Full Time	971 (67.6%)
Employed Part Time	199 (13.9%)
Unemployed	253 (17.6%)
Unemployed	115 (8%)
Not in Labor Force	138 (9.6%)
Parent Ethnicity	
White	992 (69.1%)
Asian	261 (18.2%)
Latin American	45 (3.1%)
Black	47 (3.3%)
Indigenous/First Nations	21 (1.5%)
Multiracial/Other	59 (4.1%)
Child Age—*M* (*SD*)	7.9 (1.7)
Child Gender	
Female	333 (46.4%)
Male	383 (53.3%)
Non-Binary/Unknown	0
Child Ethnicity	
White	481 (67%)
Asian	115 (16%)
Latin American	14 (1.9%)
Black	23 (3.2%)
Indigenous/First Nations	9 (1.3%)
Multiracial/Other	66 (9.2%)
Child Disability Status	
Diagnosed with Disability	88 (12.3%)
Not Diagnosed with Disability	628 (87.5%)

Notes. The numbers provided do not add up to the total of 718 (100%) due to missing data.

**Table 2:** Study variable means and counts.

Variable	*M* (*SD*)
% Time Spent Homeschooling	45.5 (36.3)
Child Externalizing Symptoms	6.8 (4)
Child Internalizing Symptoms	5.3 (4.2)
Parent Anxiety	10.3 (8.5)
Parent Depression	12.5 (10.1)
Parent Stress	12.7 (5.1)
	***n***
Live Online Class	
Yes	357
No	361
Pre-recorded Online Class	
Yes	89
No	629
Home Learning Pack	
Yes	178
No	540
Parent Employment	
Both Employed	489
One or Both Unemployed	207
Child Internet	
Reliable	460
Unreliable	257

Note. The numbers provided do not add up to the total of 718 due to missing data.

**Table 3:** Total effects of cumulative time spent homeschooling on mental health outcomes.

	Cumulative Time Spent Homeschooling				
Predictors	Est.	*t*	LLCI	ULCI	*p*
Child Externalizing	0.01 (<0.01)	1.12	−0.003	0.013	0.26
Child Internalizing	*** 0.02 (<0.01)	4.39	0.01	0.026	<0.001
Parent Anxiety	* 0.02 (0.01)	2.12	0.001	0.036	0.03
Parent Depression	** 0.03 (0.01)	2.82	0.009	0.05	<0.01
Parent Stress	0.01 (0.01)	1.16	−0.005	0.018	0.246

Note. This table shows the total effects of cumulative time spent homeschooling (X) on parent and child mental health outcomes (Y) from the mediation analysis. There were positive, significant relationships for child internalizing symptoms and parent depression and anxiety. * *p* < 0.05, ** *p* < 0.01, *** *p* < 0.001.

**Table 4:** Ordinary least squares regressions with educational resources from school as moderators.

	Child Externalizing			Child Internalizing			Parent Anxiety			Parent Depression			Parent Stress		
Predictor	Est (*SE*)	*t*	*p*	Est (*SE*)	*t*	*p*	Est (*SE*)	*t*	*p*	Est (*SE*)	*t*	*p*	Est (*SE*)	*tl*	*p*
Live Class															
Intercept	0.12 (0.17)	0.71	0.477	0.18 (0.18)	0.98	0.326	0.39 (0.37)	1.04	0.298	0.46 (0.44)	1.05	0.294	0.06 (0.22)	0.287	0.774
CTSH	<0.01 (0.01)	0.23	0.82	** 0.02 (0.01)	3.2	0.001	0.02 (0.01)	1.58	0.115	* 0.03 (0.01)	2.26	0.024	<0.01 (0.01)	0.392	0.695
Live Class	0.56 (0.33)	1.7	0.092	0.34 (0.36)	0.94	0.35	0.18 (0.74)	0.24	0.808	0.01 (0.88)	0.007	0.994	0.68 (0.43)	1.577	0.115
Interaction	−0.02 (0.01)	−1.74	0.083	* −0.02 (0.01)	−2.46	0.014	* −0.05 (0.02)	−2.58	0.01	** −0.06 (0.02)	−2.62	0.009	−0.01 (0.01)	−0.665	0.506
Pre-recorded Class															
Intercept	0.06 (0.26)	0.23	0.82	0.45 (0.31)	1.45	0.147	0.06 (0.57)	0.11	0.914	0.39 (0.71)	0.55	0.586	−0.04 (0.37)	−0.116	0.908
CTSH	<0.01 (0.01)	0.03	0.979	0.01 (0.01)	1.24	0.215	0.02 (0.02)	1.11	0.267	0.03 (0.02)	1.2	0.247	0.01 (0.01)	0.536	0.592
Pre-recorded Class	0.1 (0.52)	0.19	0.849	1.15 (0.63)	1.83	0.068	0.25 (1.14)	0.22	0.827	1.04 (1.42)	0.73	0.465	−0.1 (0.74)	−0.131	0.896
Interaction	−0.01 (0.02)	−0.63	0.527	−0.01 (0.02)	−0.52	0.604	0.01 (0.04)	0.23	0.821	−<0.01 (0.05)	−0.05	0.963	<0.01 (0.03)	0.068	0.946
Learning Packs															
Intercept	0.12 (0.18)	0.1	0.55	0.21 (0.2)	1.05	0.296	0.49 (0.38)	1.28	0.201	0.46 (0.46)	0.99	0.322	0.25 (0.24)	1.0146	0.311
CTSH	<0.01 (0.01)	0.08	0.936	0.01 (0.01)	1.66	0.098	0.01 (0.01)	0.54	0.593	0.01 (0.01)	1	0.316	<0.01 (0.01)	0.072	0.943
Learning Packs	0.23 (0.36)	0.65	0.517	0.35 (0.4)	0.89	0.377	* 1.54 (0.77)	2	0.046	1.09 (0.92)	1.18	0.238	0.77 (0.48)	1.591	0.112
Interaction	−0.01 (0.01)	−1.3	0.196	** −0.03 (0.01)	−2.95	0.003	−0.03 (0.02)	−1.26	0.209	−0.05 (0.03)	−1.92	0.055	−0.01 (0.02)	−0.885	0.377

Note. These results from the ordinary least squares regressions show that Cumulative Time Spent Homeschooling (CTSH) interacted significantly with live online classes to predict child internalizing symptoms and parent anxiety and depression and interacted significantly with home learning packs to predict child internalizing symptoms. * *p* < 0.05, ** *p* < 0.01, *** *p* < 0.001.

**Table 5:** Simple slopes analysis.

	Child Externalizing			Child Internalizing			Parent Anxiety			Parent Stress		
Moderator	Est (*SE*)	*t*	*p*	Est (*SE*)	*t*	*p*	Est (*SE*)	*t*	*p*	Est (*SE*)	*t*	*p*
Live Classes												
No	—	—	—	0.03 *** (0.01)	3.98	<0.001	0.04 ** (0.02)	2.88	0.004	0.06 *** (0.02)	3.34	<0.001
Yes	—	—	—	<0.01 (0.01)	0.53	0.599	−0.01 (0.01)	−0.73	0.469	<−0.01 (0.02)	−0.27	0.792
No	—	—	—	0.02 *** (0.01)	4.86	<0.001	—	—	—	—	—	—
Yes	—	—	—	−0.01 (0.01)	−0.74	0.462	—	—	—	—	—	—
Employment												
Unemployed	−0.01 (0.01)	−1.47	0.141	0.01 (0.01)	1.09	0.276	—	—	—	—	—	—
Employed	0.01 * (0.01)	2.44	0.015	0.02 *** (0.01)	4.46	<0.001	—	—	—	—	—	—
Internet Access												
Unreliable	—	—	—	<0.01 (0.01)	0.32	0.748	—	—	—	—	—	—
Reliable	—	—	—	0.02 *** (0.01)	4.2	<0.001	—	—	—	—	—	—

Note. These results from the simple reveal the relationship between cumulative time spent homeschooling and parent and child mental health outcomes at each level of the moderator. * *p* < 0.05, ** *p* < 0.01, *** *p* < 0.001.

**Table 6:** Ordinary least squares regressions with parent employment and child internet access as moderators.

	Child Externalizing			Child Internalizing			Parent Anxiety			Parent Depression			Parent Stress		
Predictor	Est (*SE*)	*t*	*p*	Est (*SE*)	*t*	*p*	Est (*SE*)	*t*	*p*	Est (*SE*)	*t*	*p*	Est (*SE*)	*t*	*p*
Employment															
Intercept	−0.04 (0.17)	−0.21	0.837	−0.11 (0.16)	−0.69	0.493	−0.19 (0.35)	−0.53	0.595	−0.13 (0.42)	−0.316	0.752	−0.13 (0.23)	−0.57	0.57
CTSH	<0.01 (<0.01)	0.21	0.833	*** 0.02 (<0.01)	3.76	<0.001	0.02 (0.01)	1.88	0.061	* 0.03 (0.01)	2.36	0.018	0.02 (0.01)	0.85	0.395
Employment	0.26 (0.34)	0.75	0.455	* 0.64 (0.33)	1.95	0.052	0.78 (0.71)	1.1	0.271	0.76 (0.85)	0.9	0.368	0.42 (0.46)	0.92	0.36
Interaction	** 0.02 (0.01)	2.61	0.009	* 0.02 (0.01)	2.12	0.035	0.02 (0.02)	0.81	0.416	0.03 (0.02)	1.45	0.147	0.02 (0.01)	1.88	0.061
Internet Access															
Intercept	0.24 (0.15)	1.55	0.121	0.33 (0.16)	2.03	0.042	0.23 (0.33)	0.7	0.486	0.48 (0.39)	1.24	0.217	0.29 (0.18)	1.56	0.119
CTSH	<0.01 (0.01)	0.07	0.941	* 0.01 (0.01)	2.54	0.011	0.02 (0.01)	1.48	0.139	0.02 (0.01)	1.68	0.094	<0.01 (0.01)	0.24	0.813
Internet Access	*** −1.56 (0.31)	−5.1	<0.001	*** −2.1 (0.32)	−6.49	<0.001	* −1.6 (0.67)	−2.39	0.017	*** −3.09 (0.78)	−3.94	<0.001	*** −1.88 (0.37)	−5.1	<0.001
Interaction	0.01 (0.01)	1.26	0.207	* 0.02 (0.01)	2	0.046	0.01 (0.02)	0.52	0.601	0.03 (0.02)	1.38	0.168	0.01 (0.01)	1.17	0.242

Note. These results from the ordinary least squares regressions show that cumulative time spent homeschooling (CTSH) interacted significantly with child internet access to predict child internalizing symptoms and interacted significantly with parent employment to predict child externalizing and internalizing symptoms. * *p* < 0.05, ** *p* < 0.01, *** *p* < 0.001.

## Data Availability

The data presented in this study are available on request from the corresponding author due to ethical reasons.
